# Impact of confrontation to patient suffering and death on wellbeing and burnout in professionals: a cross-sectional study

**DOI:** 10.1186/s12904-024-01393-8

**Published:** 2024-03-15

**Authors:** Anne-Catherine Delafontaine, Royce Anders, Bernard Mathieu, Cornelia Rolli Salathé, Benjamin Putois

**Affiliations:** 1 Faculty of Psychology, Swiss Distance Learning University, Technopôle 5, Sierre, 3960 Switzerland; 2 Department of Psychology, Department of Psychology, Epsylon Laboratory UR4556, University Paul Valéry Montpellier 3, Montpellier, 34000 France; 3 Chair of palliative psychology, Lausanne University Hospital and University of Lausanne, Hôpital Nestlé, Av. Pierre-Decker 5, Lausanne, 1011 Switzerland; 4 Department of Psychology, University of Fribourg, Fribourg, 1700 Switzerland; 5 Lyon Neuroscience Research Centre, CNRS UMR 5292 - INSERM U1028, Lyon, France

**Keywords:** Healthcare professionals, Palliative care, Oncology, Burnout, Psychological distress, Death confrontations, Work wellbeing, Meaning at work, Personality, Self-esteem

## Abstract

**Background:**

Palliative care and oncology generate a risk of burnout and psychological distress in professionals. The purpose of this study is to identify both psychopathological and positive factors related to mental health at work. It aims (i) to explore the extent to which these professionals are confronted with suffering, illness, and death; and to explore the prevalence of psychological distress and/or burnout, (ii) to identify potential determinants of burnout and psychological wellbeing at work, (iii) to develop an integrative model of mental health; and to identify frequency and impact of confrontations with death, and (iv) to identify profiles of professionals are at risk of developing a mental health disorder or, conversely, characterized by wellbeing.

**Methods:**

A cross-sectional questionnaire study was conducted in palliative care and oncology evaluating confrontations with death, coping, burnout, psychological distress, personality, self-esteem, well-being and meaning at work. Regressions, clustering, and structural equation modeling analyses were performed.

**Results:**

109 professionals participated (58% from oncology and 42% from palliative care), of which 79% were female, and 65% were between 30 and 49 years old. Aim i: 30% witnessed an intolerable suffering at least 9 times a month, 45% reported moderate to high levels of burnout, 39% suffered from anxiety and 11% from depression. Aim ii: the determinants of burnout were the personality traits conscientiousness and neuroticism, low meaning of work, and low wellbeing (R^2^ = 0.44). The determinants of wellbeing were work meaning, depersonalization, self-esteem, fulfillment and low emotional exhaustion (R^2^ = 0.71). Aim iii: the integrative model included both well-being (self-esteem, conscientiousness) and psychopathology (neuroticism, anxiety) parameters, and strongly satisfied the standard SEM goodness of fit indices (e.g., CFI, IFI, and TLI ≥ 0.95). Aim iv: three profiles were identified: (a) a “distressed profile” with a majority of professionals at the patient’s bedside, (b) a “disengaged profile” with professionals working as second-line consultants, (c) a “wellbeing profile” contains profiles of caregivers insensitive to psychological distress and with a high level of positive Impact of confrontation on different areas of their lives.

**Conclusions:**

An integrative approach is essential to understand the full range of mental health issues for professionals. Meaning of work is a key factor in professional interventions that should primarily affect front-line professionals with limited experience.

**Supplementary Information:**

The online version contains supplementary material available at 10.1186/s12904-024-01393-8.

## Introduction

Healthcare professionals working in palliative and oncological care are daily confronted with serious illnesses, or the suffering and death of patients, which can leave marking impressions. Indeed, dealing with the loss of patients has been recognized as one of the most challenging demands of clinical practice careers to date [1, 2]. Other sources of stress in the profession are as noteworthy, such as the stress involved in delivering bad news [3], relieving difficult patient symptoms [4], or arbitrating complicated family relations [5, 6]. These challenges provide some central examples for which the increased risk of burnout that has been identified in healthcare careers could be explained.

The problem of burnout spurs numerous repercussions in the field as it has been associated with poorer physical and mental health [7, 8], lower quality of patient care [9, 10], more medical errors [11, 12], lower empathy [13], employee absenteeism, and turnover [14]. Prevalence rates of burnout and its precursors in the sector can be considered worrisome. A recent meta-analysis in oncological care reported 30% emotional exhaustion rates in nurses [15] and 32% in physicians [16]. A systematic review in palliative care revealed prevalence rates of 17% for burnout, in which nurses scored higher in emotional exhaustion (19%) and depersonalization (8%), and physicians scored lower in their sense of personal accomplishment (41%) [17]. Despite these career challenges and pathological rates, it is remarkable to note that in various studies as many as 70% professionals have reported limited degrees of emotional exhaustion. This begs the questions, *why and what can be learned to better protect the remaining third of professionals who are vulnerable to such pathologies*?

Previous research has identified the risk of burnout as not only linked to work context and degree of workload [18], but also to individual factors such as personality trait [19], self-esteem [20], and mental or affective disorders such as depression [21]. In fact, the relationship between burnout and depression among nurses has been well documented [22]. Furthermore, several studies evaluating professionals in oncological and palliative care demonstrated that certain personality traits, such as a higher level of openness, conscientiousness, and extraversion are linked to a higher sense of professional accomplishment and lower levels of burnout [23, 24]. Moreover, higher levels of global self-esteem [25] and meaning of work [5] were identified as protective factors against burnout in nurses; the latter factor serving as the ability to derive existential meaning from one’s work execution, experiences, and purpose [26]. These psychological and individual factors may explain in part, why a large proportion of caregivers are not, or are only minimally psychologically impacted, by regular exposure to patient suffering and death.

Research addressing pathologies like burnout and depression significantly increased over the last twenty years, especially in line with emergent frameworks such as salutogenesis [27–29] and positive psychology [30]. These frameworks place additional emphasis on considering positive health factors, which for example, could be crucially protective. Indeed, they advocate that research on conditions and processes contributing to an optimal functioning of individuals, groups, and institutions [31] is just as important as researching illness and pathology. Furthermore, they could play an essential role in addressing professional pathologies through prevention.

Psychological wellbeing at work can be defined as a positive work experience that engenders life satisfaction, confidence, and contentment, developed by individual and social relations at work [32]. Addressing the wellbeing of healthcare professionals at work is not only important on an individual level, but also on various organizational and societal levels, as professional wellbeing has been found to influence work engagement, performance [33], and employee retention. Considering society’s large aging population, improved wellbeing at work for better employee retention could crucially respond to the worrisome lack of professionals in palliative career to treat them. Research on wellbeing at work has demonstrated so far that self-esteem [34], as well as the personality traits conscientiousness, extraversion, and agreeableness [35], predicted improved subjective wellbeing, while neuroticism predicted the opposite [35]. In addition, the meaning of work, notably as an intrinsic motivator [36], positively influenced the employee’s affective commitment to an organization, as well as the psychological wellbeing at work [26]. In this respect, as noted by Moreno-Milan et al. [5] p. 3: “health professionals who have a greater sense of the meaning of their work are able to recognize its importance, thus changing how they interpret certain critical situations. Exposure to patient distress may induce less stress in healthcare professionals who find a greater meaning in such events”. Also, recent research may suggest several important elements that may be complimentary to this protective angle, such as human relationships, death acceptance, and one’s sense of dignity [2].

Regarding the career of healthcare professionals, no previous study can be identified to our knowledge that simultaneously assessed the protective factors against mental disorders and those for wellbeing at work. In this respect, the present study aimed for an integrative approach through a multifactorial analysis of work context variables, personality traits, self-esteem, work meaning, and confrontation dynamics, to determine wellbeing outcomes in healthcare professionals repeatedly facing patient suffering and death. Such integrative approaches have been encouraged notably within the framework of second wave positive psychology: PP 2.0 [37–40]. This nuanced approach assesses complex interactions not only by considering positive factors and individual resources, but also by integrating parts of the dark, yet inseparable, side of human experiences, such as loss and suffering. Indeed, the human experience of regular confrontation to patient suffering and death in oncological and palliative care presents a unique opportunity to study such experiences in a more holistic manner. Furthermore, the potential to gain a better understanding of the interplay between positive and negative factors in the work context can contribute to improved employee-wellbeing programs establishing an appropriate work-life balance. So far and only recently, this notion has been investigated formally in respect to burnout [41].

The principal objectives of the present study are to (i) explore the extent to which healthcare professionals who are regularly confronted with suffering, illness, and death are stricken with psychological distress and/or burnout; (ii) identify potential determinants of burnout and psychological wellbeing at work in healthcare professionals; (iii) develop a comprehensive and integrative model of wellbeing at work for healthcare professionals regularly confronted with death and suffering, as a preliminary model that can be refined in further researches; and finally (iv) identify profiles of healthcare professionals who are particularly at risk of developing a mental health disorder or, conversely, possess a robust wellbeing, which could be used in future preventative or targeted employee wellbeing programs. We hypothesized that a strong meaning of work, high self-esteem, select personality factors, low predisposition of anxiety and depression, and the ability to interpret the end of life positively, are the most protective factors against burnout and coincide as determinants of wellbeing at work.

## Methods

### Participants

#### Sampling method

The target population consisted of participants working regularly in the oncological and palliative care services in a hospital setting. In order to sample from this population, the researchers presented the research project to the oncological and palliative services management staff, in the region’s university hospital as well as employees during their respective team meetings. Then, the department heads provided a list of names to the researchers, which specified the oncology and palliative care employees who were in regular contact with patients, along with their contact information (email) and position title. In total, the resultant list consisted of *n* = 260 potential participants.

#### Inclusion and exclusion criteria

The inclusion criteria consisted of the following: (i) being 18 years or older, (ii) being a healthcare professional in regular physical or telephone contact with patients (health care assistants, community health care assistants, nurses, physicians, spiritual advisors, psychologists, and administrative staff), and (iii) working in palliative or oncological care. The exclusion criteria were: (i) working less than 20 h a week or (ii) being a student. Socio-demographic and occupational data were collected (specified in Sect. 2.3), making it possible to verify these inclusion criteria.

### Procedure

This cross-sectional study was conducted in the oncological and palliative care services of a university hospital, utilizing empirically-validated questionnaires. All methods were carried out in accordance with Strengthening the Reporting of OBservational studies in Epidemiology STROBE, [42]. The study was approved by the university ethics committee (Unidistance’s ethics committee, authorization number: 2019-06-00002), with the condition that there would be no comparison between the two services (palliative vs. oncological care) in this research.

The list of eligible employees provided by the department heads (potential of *n* = 260) were sent an email inviting their participation in the study. In this email, they were provided access to a document presenting the study’s purpose, accompanied by a link to complete the questionnaires online. The first page of the survey contained a declaration of consent for voluntary participation in the study. It stated that the data would only be used in an anonymous form. It was also specified that participants could revoke their consent to take part at any time, without having to justify their decision, and that they could withdraw from the study at any time, without having to justify their decision, and, if necessary, request that their data be destroyed. In addition, authorization was given to use the anonymized data collected for scientific purposes and to publish the results of the research in scientific journals. The last line of the form was: “By clicking on “CONTINUE”, I agree to take part in the study and take note that the click acts as my signature”. The average completion time of the study was 20 min. The study was presented on numerous occasions at team meetings in the departments concerned, and invitation emails were sent out. The data collection took place between June and August 2019.

### Measures

**Socio-demographic and occupational data** were collected, specifically: profession, professional role, number of hours worked per week, years of experience, age were asked, making it possible to verify the inclusion criteria. Other questions such as gender, number of years working in current position, living as a couple and number of dependent children were optional, to avoid any possible identification of candidates.

**Burnout** was measured with the French validated *Maslach Burnout Inventory (MBI-HSS)* from Maslach and Jackson [43], a 22-item questionnaire that measures three components of burnout: emotional exhaustion (9 items, higher scores reflect higher exhaustion), depersonalization (5 items, higher scores reflect higher depersonalization) and personal accomplishment (8 items, lower scores reflect lower achievement). Burnout positivity is determined by the MBI via either of two joint conditions: (1) when both subscales Exhaustion and Depersonalization are > 17, or (2) when Exhaustion is > 17 and sense of Accomplishment is < 35. Cronbach’s alpha was *α* = 0.73 for this questionnaire.

**Wellbeing at work** was measured with the *Index of Psychological Wellbeing at Work (IPWW)* [44] a 25-item questionnaire developed in French that measures five subscales, each composed of 5 items on a Likert scale that are averaged in order to obtain the composite score. A higher composite score in each subscale reflects higher work wellbeing. These five components are: interpersonal fit, job satisfaction, sense of work competence, perceived recognition, and willingness to commit. Cronbach’s alpha was *α* = 0.94 for this questionnaire.

**Psychological distress** was examined with the French validated *Hospital Anxiety and Depression Scale (HADS)* [45], a 14-item questionnaire rated on a Likert scale yielding a total score (0–42), composed of an anxiety score (0–21) and a depression score (0–21), with higher scores reflecting higher distress. The usual thresholds were utilized for this scale: an anxiety score of ≥ 8 or a depression score of ≥ 8 determine positivity for the respective problematic. Cronbach’s alpha was *α* = 0.81 for this questionnaire.

**Self-esteem** was measured with the French validated *Rosenberg’s Self-Esteem Scale (RSE)* [46] a 10-item questionnaire, rated on a Likert scale yielding a total score with higher scores reflecting higher self-esteem. Cronbach’s alpha was *α* = 0.81 for this questionnaire.

**Work meaning** was measured with the *Meaningfulness Scale (MS)* from Spreitzer [47–49], a 6-item questionnaire rated on a Likert scale yielding an average total score, with higher scores reflecting higher sense of work meaning. Alpha coefficient reliabilities of between 0.90 and 0.93 have been reported for scores from the PMS. The reliability of scores from the PMS with their study sample was 0.95. We utilized the French translation provided by Desgroseilliers [50]. Cronbach’s alpha was *α* = 0.91 for this questionnaire and the standard confirmatory factor analysis (CFA) goodness of fit indices showed strong values for a 1 factor solution (GFI, AGFI, CFI, TLI > 0.95).

**Personality** was measured with the French validated *Ten Item Personality Index (TIPI)* [51, 52]. This 10-item questionnaire measures the five factors of the Big Five personality traits, with 2 items for each dimension that are averaged. Openness refers to new ideas and experiences; Conscientiousness refers to self-discipline and organization; Extraversion refers to enthusiasm and sociability; Agreeableness corresponds to being tolerant and warm in interpersonal relationships; and Emotional Stability, which is considered the opposite of neuroticism. The higher the score, the more the personality trait is present. The Cronbach *α*’s were 0.68, 0.40, 0.50, 0.73, and 0.45 respectively.

**Confrontation to suffering and death**, its **impact on psychosocial variables**, and **coping strategies** that are specifically relevant to the healthcare work environment were measured with a *Confrontation to Suffering and Death Questionnaire (CSDQ)*, developed for the purpose of this study with the objective of being appropriately adapted to the healthcare sector. This questionnaire CSDQ has 17-item questions on a Likert scale with three subjects:

I. *The degree of confrontation to suffering and death* which is measured by six questions that were based on a review of the palliative care literature on risk factors for healthcare worker professional burnout in relation to patients or their relatives [5, 6]. For these six questions, the participant has to indicate the frequency of exposition within the last month to the following statements: (i) being informed of treatment failure or death of a patient, (ii) witnessing the death of a patient, (iii) the number of patients who have died while under their personal care, witnessing the intolerable suffering of (iv) a patient, (v) a family, or (vi) an agonizing patient. The response to each question was indicated on a 1–4 Likert scale. The composite score is calculated as the sum of the six questions, with higher scores reflecting a higher overall degree of confrontation to suffering and death (Cronbach’s alpha was *α* = 0.88).
II. *The impact of suffering and death on work-related psychosocial variables* which is measured by six questions on a scale from − 8 (very strong negative) to 8 (very strong positive) impact. With this scale and in respect to the last month, the participant rated the effect that confrontation to suffering and death had on the following six domains: their investment in (i) patient relationships, (ii) personal relationships, and (iii) leisure activity, their (iv) sense of usefulness/utility at work, and finally their personal representation (v) of life and (vi) death. The total score was computed by the sum of the six items (–48 to 48), higher scores reflecting more positive impact (Cronbach’s alpha was *α* = 0.81).
III. *The use of different coping strategies to deal with suffering and death* which is measured by five questions on a Likert scale of 1–6, in which the participant reported the extent to which they utilized the following coping strategies to ease handling these regular confrontations of patient suffering and death: (i) exchanging with supervisors, (ii) sharing with colleagues, (iii) talking to a relative, (iv) consulting with a psychotherapist, and (v) other supports. The total score was calculated as the sum of the five items, with higher scores reflecting higher coping supports (Cronbach’s alpha was *α* = 0.48).

Each subscale was reviewed and tested by a group of physicians and nurses who were specialized in either oncological or palliative care. However, we would like to point out that the present data is inadequate to conduct a thorough evaluation of the psychometric properties of the CSDQ. The initial structure of the questionnaire was assessed through an Exploratory Factor Analysis (EFA) method (see Supplementary Materials, Table S1, that provides the detailed results). Bartlett’s test of sphericity (*p* < .05 preferred) and the Kaiser-Meyer-Olkin (KMO) measure of sampling adequacy (KMO ≥ 0.60 preferred) were verified to assess the appropriateness of performing factor analysis on the data, here satisfied with *p* < .001 and KMO = 0.67 respectively. Then, a scree plot analysis was assessed that verified the support of a latent 3-factor structure of the CSDQ (available as Figure S1 in the Supplementary Materials).

The EFA resulted in a three-factor structure where the items loaded most strongly on their respected factors, and in which the factors explained 19.8%, 16.0%, and 6.4% of the variance respectively, with a total of 42.4%. Generally, explaining 40% or more of the variance is considered robust [53]. The final score reflects that the way in which individuals may engage in social coping strategies is heterogeneous. Confirmatory analyses on a larger sample would be necessary to validate the structure of this questionnaire.

### Statistical analyses and modeling

The analytical approach utilized a similar methodology as used in [54]. First, descriptive statistics and frequency counts (e.g. positivity) were calculated along the relevant variables and questionnaires (see Tables S2 and S3 in the Supplementary Materials). Then, in preparation to satisfy modelling and statistical criteria (e.g., normality, homoskedasticity, linearity for regression), noncategorical variables were normalized via the Yeo-Johnson transformation [55] and scaled.

Then, backward stepwise linear multiple regression models (*statsmodels* package in Python, version 0.14) were realized to identify the most pertinent variables that predict the three component subscales of professional burnout (exhaustion, depersonalization, and accomplishment, MBI-HSS), as well as psychological wellbeing at work (IPWW) respectively. For each application, first in the full model with all variables, approximately 5% of the data were filtered as outliers based on Cook’s Distance [56] values that exceeded the 95% quantile. Then, variables in the model were eliminated recursively by which removal would most improve the Akaike Information Criterion, AIC [57]. For both resultant models, the necessary assumptions and diagnostics were rigorously evaluated, and these are provided in the Results section.

Thirdly, path analysis was performed within the structural equation modeling (SEM) framework in order to obtain an integrative model of work wellbeing and mediated relationships (*lavaan* package in R). No latent explicative/composite variables were added into the model. The resultant networks were obtained through a data-driven approach: optimizing a Bayesian network structure learning algorithm (*bnsl* package in R). In each case, the simplest SEM network structure that appropriately satisfied the standard reference SEM diagnostics was selected. Then, the plausibility of several additional paths was evaluated (e.g., theoretically motivated, or suggested by the standard modification indices provided through the *lavaan* package), and these were retained only if they substantially improved the SEM diagnostics. The performance diagnostics of the final model, along at least 10 criteria, are provided in the Results section.

Fourthly, a principal components analysis and *k*-means clustering of the participants was performed (*scikit-learn* library in Python) to obtain additional insights into the healthcare worker sample. The observed Hopkins *H* = 0.65 for the data [58, 59], which supports a tendency to cluster. Specifically, the principal component decomposition of the data (*N* = 6 dimensions) was submitted to a *k*-means algorithm [60, 61]. An optimal clustering result that balanced interpretable parsimony and performance (cluster separation and compactness) was obtained at *K* = 3 clusters. The performance diagnostics for this final model are provided in the Results section.

## Results

### Description of the healthcare worker sample

A total of 139 healthcare professionals participated in the study (53% response rate), of which 109 professionals met the inclusion criteria (one person working less than 20 h per week was excluded) and answered the questionnaire completely (42% response rate). The majority of the participants were aged 30 to 49 years (65%), otherwise 16% and 19% were 19–29 and 50–65 years old respectively; 21% were male. A fairly balanced representation was present in the sample between the oncological (58%) and palliative (42%) care services. With regard to the service line, over half of the participants worked in the first line of care (58%, i.e., working at the bedside), one third in the second line (29%, i.e., working as nurse consultants) and over 10% in the third line (13%, i.e. administrative staff or researchers). Occupationally, 60% of participants were caregivers and nurses (67% of which were in the first line of care, 33% in the second), 28% were physicians (66% first line, 34% second), and 13% consisted of other healthcare professionals all working in the third line.

### Prevalence of confrontations with suffering and death, their impact and ways of coping, as well as prevalence of psychological distress and burnout

Based on the three MBI subscales scores, 15.7% of participants can be considered as clinically positive for burnout (see the criteria in the Methods). On the MBI emotional exhaustion subscale, 32.1% of participants satisfied the criteria for severe exhaustion (score ≥ 30), and 45.0% for moderate exhaustion (30 > score ≥ 18). A number of the participants also satisfied the HADS criteria to be considered positive for clinical anxiety (38.9%, score ≥ 8) and clinical depression (11.1%, score ≥ 8).

Based on the CSDQ questionnaire, it is particularly noteworthy that 30% of the professionals who participated report having witnessed intolerable suffering in the patient on more than nine occasions during the last month, and 25% report having witnessed intolerable suffering in a family member of the patient on more than 9 occasions during the last month (see Table 1).

**Table 1:** Proportion of professionals facing confrontations with death and suffering during the last month (*n* = 109)

	None (%)	1–4 times (%)	5–8 times (%)	9 or more times (%)
Witnessed intolerable suffering of a patient	7	40	23	30
Witnessed intolerable suffering of a family member	11	46	18	25
Gave care to a patient in agony	33	31	11	25
Witnessed the death of a patient	69	18	4	9
Gave care to a patient who passed away	20	38	18	24
Participated in announcing bad news	24	39	18	19

It is interesting to note that the majority of them report that these confrontations have had a very positive influence on their representations of existence (83%) and of death (70%), but also on the perceived usefulness of their work (68%) and their relational investment with patients (65%) (see Table 2).

**Table 2:** Proportion of professionals having positive impact of confrontations on personal and professional levels (*n* = 109)

	Weak to moderate impact (%)	Strong to very strong impact (%)
Positive representations about life	17	83
Positive representations about death	30	70
The usefulness of work	32	68
Investment in patient relations	35	65

Concerning the support used by the professionals to face death and suffering, informal support (sharing with colleagues and talking with relatives) was the most frequently mentioned one by the professionals: 65% used it at least once per week and 82% daily (see Table 3).

**Table 3:** Proportion of professionals using different coping strategies (*n* = 109)

	None or rarely (%)	1–4 times a month (%)	Every day (%)
Sharing with colleagues	11	39	50
Talking to a relative	42	26	32
Talking to supervisors	55	41	4
Seeing a psychotherapist	86	14	0
Other strategies	96	2	2

### Determinants of burnout

In order to identify the significant predictors of each of the three MBI-HSS subscales that combine to determine professional burn-out, a statistical modelling was performed using linear regression. For each of the individual models, the diagnostics for appropriate model fit were satisfied (e.g., see [62–64]). Residual normality was assessed and satisfied by the Komolgorov-Smirnov Jarque-Bera, Anderson-Darling, and Omnibus tests (all *p* > .05); homoskedasticity by the Breusch-Pagan, White, and Goldfeld-Quandt tests (all *p* > .05). The absence of multicollinearity was verified and satisfied by small observed variance inflation factors for each predictor (largest value across all models was 2.3); multivariate normality was satisfied by the Yeo-Johnson transformation; absence of autocorrelation was verified and satisfied by the Ljung-Box and Lagrange Multiplier tests (both *p* > .05); and linearity verified and satisfied in the Rainbow and Ramsey tests (both *p* > .05).

Starting with emotional exhaustion, a significant equation was found (*F* (9, 90) = 9.52, *p* < .001) with an *R*^*2*^ and adjusted *R*^*2*^ of 0.49 and 0.44 respectively. Conscientiousness (TIPI) was the only significant predictor associated with increased sensitivity to exhaustion, while emotional stability (TIPI), work meaning (MS), work wellbeing (IPWW), and positive impact of death confrontation on leisure time (CSDQ) were significant predictors of less exhaustion (see Table 4).

Next in respect to depersonalization, a significant equation was found (*F* (8, 89) = 10.37, *p* < .001) with an *R*^*2*^ and adjusted *R*^*2*^ of 0.48 and 0.44, respectively. The degree of confrontation to suffering and death (CSDQ) was the only significant predictor of more depersonalization, while professional experience, positive impact on patient relations (CSDQ), work meaning (MS), coping strategies (CSDQ), agreeableness (TIPI), and line of service were significant predictors of less depersonalization (see Table 4).

Thirdly in respect to accomplishment, a significant equation was found (*F* (11, 86) = 7.68, *p* < .001) with an *R*^*2*^ and adjusted *R*^*2*^ of 0.50 and 0.43, respectively. Work wellbeing (IPWW) was the strongest predictor of more accomplishment, while coping strategies (CSDQ), positive impact on patient relations (CSDQ), and emotional stability (TIPI) followed. Professional experience and agreeableness (TIPI) were also significant positive predictors (see Table 4).

**Table 4:** Linear regression modelling results for the three subscales of professional burnout (MBI-HSS, *n* = 103)

		B	95% CI	t	*p*
**MBI Emotional Exhaustion**					
	Conscientiousness (TIPI)	0.18	[0.01, 0.35]	2.07	0.04
	Emotional Stability (TIPI)	-0.23	[-0.38, -0.09]	-3.15	0.002
	Work Meaning (MS)	-0.25	[-0.46, -0.05]	-2.43	0.02
	Work Wellbeing (IPWW)	-0.25	[-0.47, -0.04]	-2.32	0.02
	Positive Impact on Leisure Activity (CSDQ)	-0.17	[-0.32, -0.02]	-2.18	0.03
**MBI Depersonalization**					
	Degree of Confrontation (CSDQ)	0.16	[0.01, 0.31]	2.17	0.03
	Professional Experience	-0.28	[-0.44, -0.12]	-3.42	0.001
	Positive Impact on Patient Relations (CSDQ)	-0.22	[-0.38, -0.06]	-2.78	0.007
	Work Meaning (MS)	-0.22	[-0.38, -0.06]	-2.71	0.008
	Coping Strategies (CSDQ)	-0.2	[-0.36, -0.03]	-2.35	0.02
	Agreeableness (TIPI)	-0.18	[-0.34, -0.01]	-2.16	0.03
	Line of Service	-0.17	[-0.33, -0.01]	-2.13	0.04
**MBI Accomplishment**					
	Work Wellbeing (IPWW)	0.32	[0.11, 0.53]	3.08	0.003
	Coping Strategies (CSDQ)	0.21	[0.06, 0.37]	2.69	0.009
	Positive Impact on Patient Relations (CSDQ)	0.17	[0.02, 0.32]	2.19	0.03
	Emotional Stability (TIPI)	0.15	[0.01, 0.3]	2.06	0.04
	Professional Experience	0.15	[0.0, 0.3]	2.0	0.048
	Agreeableness (TIPI)	0.15	[-0.0, 0.31]	1.99	0.049

Note. All variables were Yeo-Johnson transformed for normality and standardised. In the interest of brevity, variables with *p*-values > 0.05 are not listed. Six participants were filtered in each regression; those exceeding the 95% quantile of Cook’s distance measures as noted in the Methods, leading to *n* = 103 from the original 109.

### Determinants of psychological wellbeing at work

Next, a statistical modelling with linear regression was also performed to identify the significant determinants of psychological wellbeing at work (IPWW). Its satisfaction of appropriate model conditions was also confirmed along the ensemble of diagnostics tests that were presented in the previous section.

A significant equation was found *F* (11, 86) = 23.90, *p* < .001 with an *R*^*2*^ and adjusted *R*^*2*^ of 0.75 and 0.72 respectively. The strongest predictors of higher psychological wellbeing at work (IPWW) were work meaning (MS), depersonalization (MBI), self-esteem (RSE), accomplishment (MBI), positive impact on patient relations (CSDQ), and line of service. In contrast, the strongest predictors of lower work wellbeing were exhaustion (MBI) and higher degree of confrontation to suffering and death (see Table 5).

**Table 5:** Linear regression modelling results for psychological wellbeing at work (IPWW, *n* = 103)

	B	95% CI	t	*p*
Work Meaning (MS)	0.46	[0.34, 0.59]	7.42	< 0.001
Depersonalization (MBI)	0.18	[0.05, 0.3]	2.82	0.006
Self-Esteem (RSE)	0.14	[0.03, 0.25]	2.56	0.01
Accomplishment (MBI)	0.15	[0.03, 0.28]	2.4	0.02
Positive Impact on Patient Relations (CSDQ)	0.12	[0.01, 0.22]	2.17	0.03
Line of Service	0.11	[0.0, 0.22]	1.99	0.049
Exhaustion (MBI)	-0.23	[-0.35, -0.1]	-3.56	0.001
Degree of Confrontation (CSDQ)	-0.16	[-0.26, -0.05]	-2.96	0.004

Note. Variables above the first line significantly predict more psychological wellbeing at work (IPWW), and below less. In the interest of brevity, variables with *p*-values > 0.05 are not listed. As in the previous regressions, six participants were filtered; those exceeding the 95% quantile of Cook’s distance measures as noted in the Methods, leading to *n* = 103 from the original 109.

### Comprehensive and integrative model of health for professionals in oncology and palliative care

**Fig. 1:**
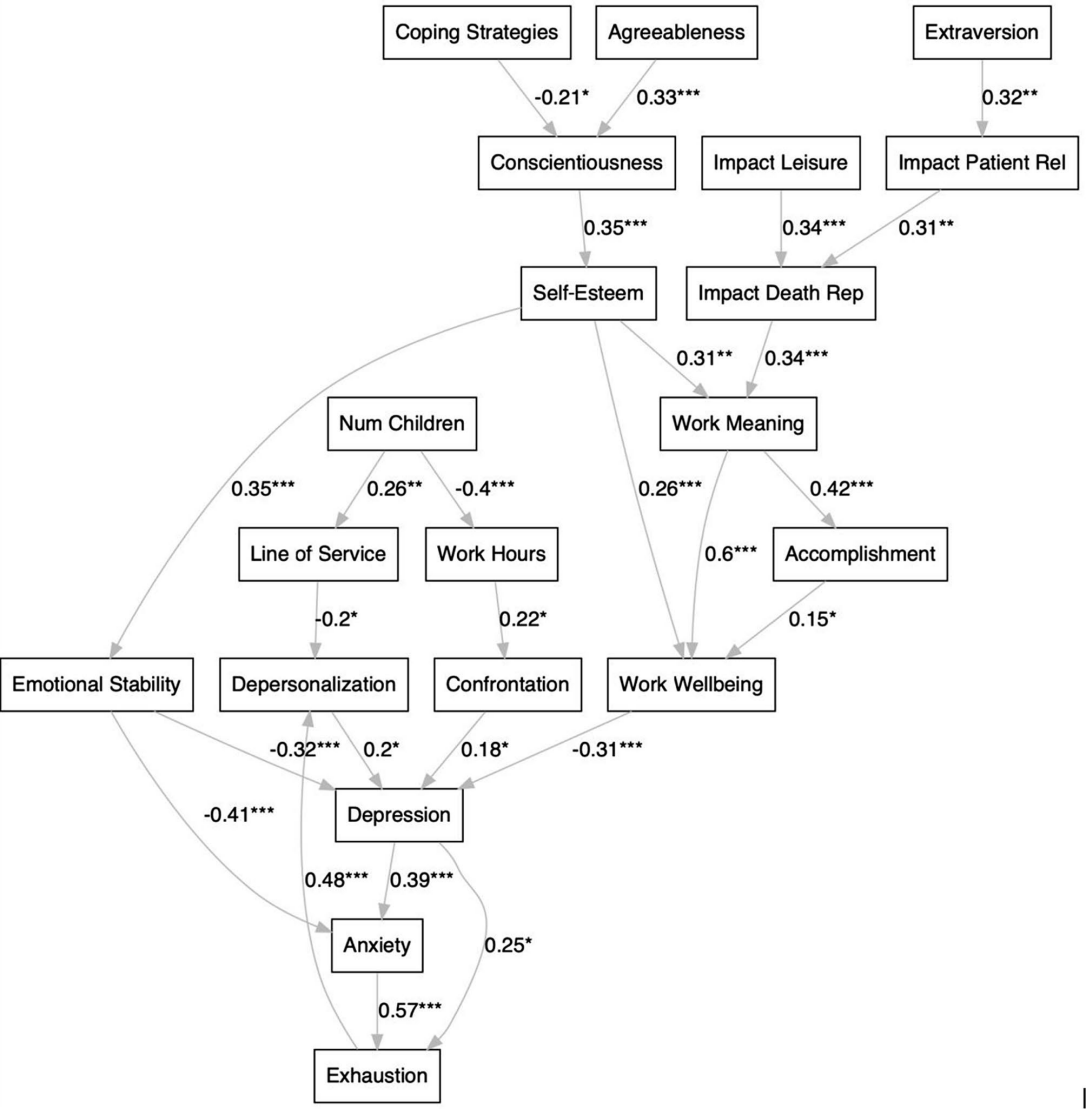
Integrative model via path analysis with the structural equation modelling framework (*n* = 109). *Note.* The model network structure was principally derived through a data-driven, Bayesian network structure learning algorithm. Significance levels of the causal relationships modelled: *** *p* < .001, ** *p* < .01 and * *p* < .05. See Methods and Table S1 in the Supplementary Materials for a more detailed description of the variables; Work Wellbeing = composite score of the IPWW subscales

Path analysis via the structural equation modelling (SEM) framework was used to develop an integrative model, provided in Fig. 1, that can reveal the intermediary relationships between and psychological wellbeing variables. The network structure of the model was obtained through a data-driven approach, specifically by the convergence of a Bayesian network structure machine learning algorithm. As provided in Table S4 in Supplementary Materials, the resultant SEM satisfied the majority of the standard fit diagnostics [65–68] specifically 8 out of 10 thresholds. Particularly, the Goodness-of-Fit (GFI), and the Adjusted Goodness-of-Fit (AGFI) were not satisfied, however these statistics have been discouraged on the basis that they are known to be strongly affected by sample size [69, 70].

Based on the SEM path analysis, it is found that higher levels of self-esteem (RSE) are associated with more work meaning (MS), accomplishment (MBI), work wellbeing (IPWW), and emotional stability (TIPI), which in-turn are associated with less psychological distress (HADS), and emotional exhaustion (MBI). Individuals with higher traits of agreeableness and conscientiousness (TIPI) tend to have higher self-esteem (RSE); and those seeking more coping strategies (CSDQ) tend to have lower conscientiousness (TIPI). Exposure to patient suffering and death in individuals with higher traits of extraversion (TIPI) tends to have a positive impact on patient relations and in-turn are more positively impacted in their representation of death (CSDQ). This latter variable, which is associated with greater work meaning (MS), is also positively predicted by impact on leisure activity (CSDQ).

Furthermore, individuals who work in the first lines of service have higher scores of depersonalization, which is associated with the triplet of depression, anxiety (HADS), and emotional exhaustion (MBI). Similarly, the extent of confrontation to suffering and death (CSDQ) is associated with this triplet, and those who work more hours tend to be confronted to a higher degree. Individuals with more children, who also tend to be older, work fewer hours, work in the later lines of service and may hence be less at risk for these negative variables. Finally, emotional exhaustion (MBI) that is predicted by depression and anxiety (HADS), may lead to additional depersonalization (MBI), which may in-turn predict more depression (HADS).

### Profiles of professionals at risk of burnout or concerned by wellbeing

**Fig. 2:**
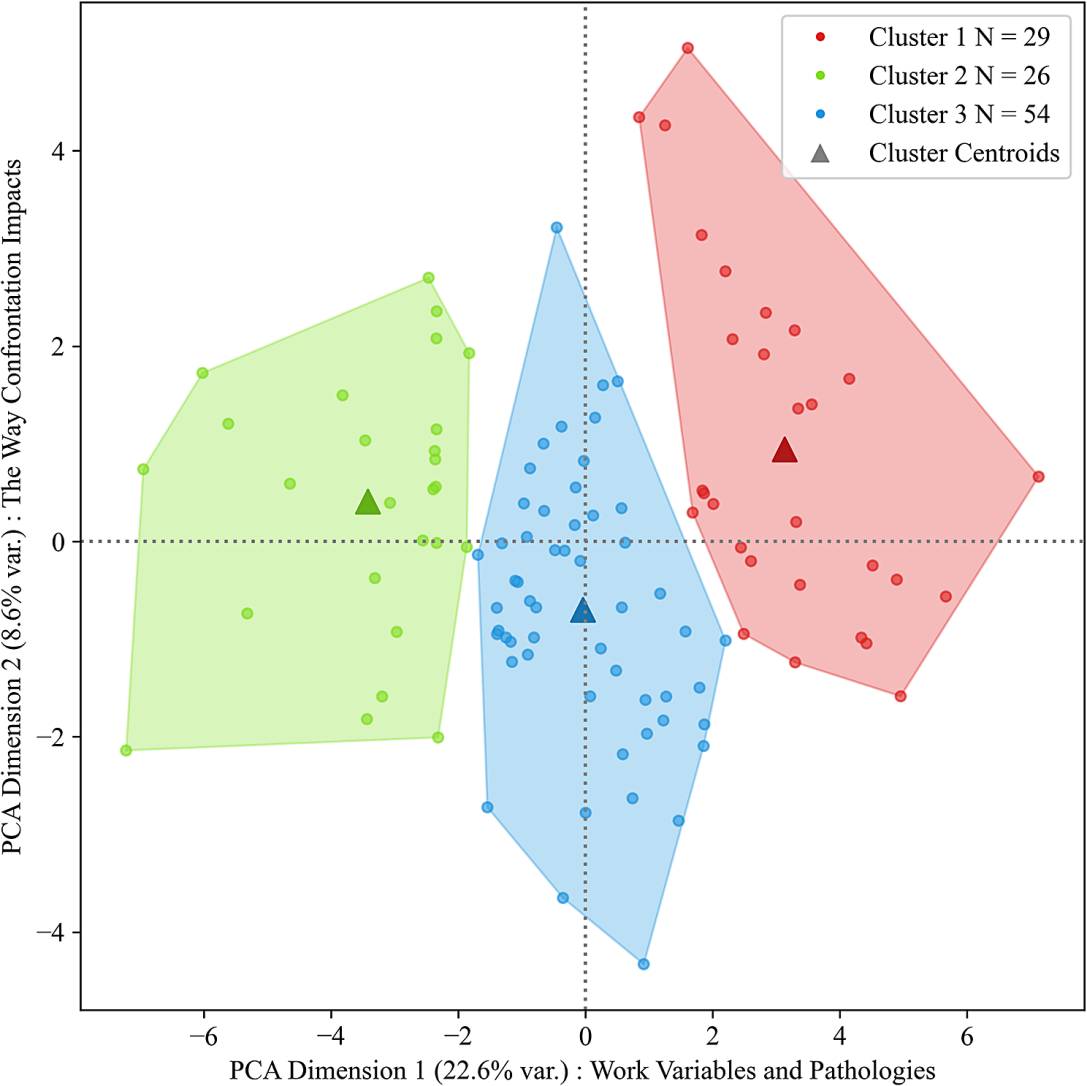
Professional clusters (*n* = 109). *Note.* Clustering Diagnostics of the k-Means algorithm results to observe the degree of within-cluster cohesion and between-cluster separation with respect to the principal dimensions of the data that explain the total variance

A clustering analysis was performed to better understand the different profiles of healthcare workers at risk of burnout or psychological wellbeing. As shown in Fig. 2, three well-defined clusters were derived from the *k*-Means algorithm analysis, which are well-separated and compact along the first two principal dimensions of the data. This appropriate result coincides with the performance on the following calculated diagnostics: the Davies-Bouldin score = 1.72 ([0, ∞+) lower values preferred, [71], Calinski-Harabasz score = 30.82 ([0, ∞+) larger values preferred [72], Dunn Index = 1.49 ([0, ∞+) larger values preferred, [73] and Silhouette score [74] was 0.16 ([-1.0, 1.0], larger values preferred). Based on an examination of the factor loadings from the principal component analysis, the first dimension consisted mainly of protective work variables (meaning, interpersonal relationships, recognition) and pathologies (exhaustion, anxiety, depression), and the second dimension consisted of the way confrontation to suffering and death impacts the individual (use of leisure activities, sense of usefulness, representation of life and death); as a reminder, these impact questions allowed for selecting on a Likert scale, either a negative or positive impact.

**Fig. 3:**
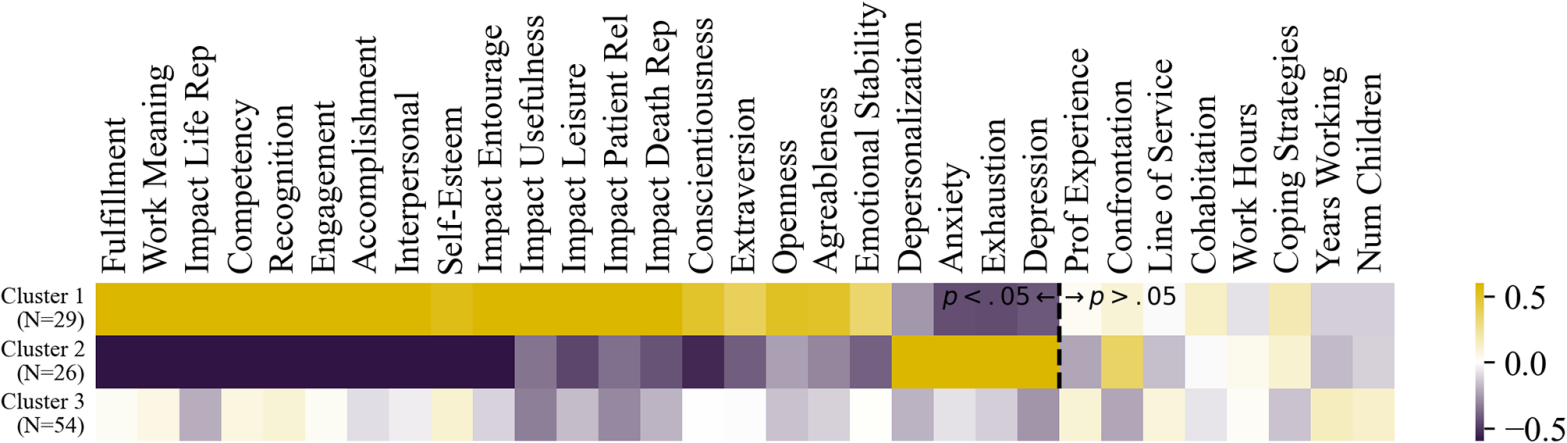
Mean values of each variable (after Yeo-Johnson transformation and standardization) for each cluster. Note. Variables are sorted based on the most significantly different positively between cluster 1 and 2, significantly different negatively, then non-significant differences. All pairwise significance tests between the clusters are provided in Table S2 in the Supplementary Materials

The cluster means for each of the variables collected are provided in Fig. 3, and they are sorted based on the strongest significant positive differences, then negative significant differences, between the first two clusters. The first two clusters bring together individuals who are equally confronted with suffering and death (Confrontation, CSDQ). The third cluster differs from the first two in that it includes individuals who are significantly less confronted with suffering and death. Among individuals identically confronted with suffering and death (Clusters 1 and 2), two mirror profiles emerge regardless of their age or professional experience: Cluster 1 contains profiles of caregivers insensitive to burnout, depression, and anxiety has a high level of Impact on Life Representation (CSDQ), Work Meaning (MS), psychological work facets (e.g., Work Fulfillment, Competency, Engagement, Recognition and Accomplishment from the IPWW), and higher Self-Esteem (RSE). Conversely, Cluster 2 contains individuals subject to burnout, depression and anxiety have a low level of positive psychology. In contrast, Cluster 3 which exhibits the lowest amount of confrontation, presents low incidence levels of burnout and psychological distress despite their lower levels of protective variables and coping, is explained by this lower exposition. Also, these individuals tend to have been working longer in the field, have children, are older, a higher Self-Esteem (than Cluster 2), and may be considered less sensitive overall based on their more moderate personality traits. For a full table of pairwise significance tests between the clusters, see Table S5 in the Supplementary Materials.

## General discussion

### Statements of principal findings

This study investigated the professional burnout and psychopathologies that occur in hospital careers and are related to employees’ regular and frequent confrontation with suffering and death. Both employee wellbeing and pathological variables were jointly recorded and analyzed via multifaceted and robust methods that allowed for a detailed analysis of the effects and interrelationships to be obtained for each of several key points of view (i.e., multivariate prediction, path analysis/mediations, and clustering).

The rate of healthcare professionals suffering from depression and anxiety symptoms was found higher here than in the general population [75]. In contrast, the rate of pathological burnout based on the MBI scale was similar to that of other studies [17]. However, crucially the rate of emotional exhaustion (MBI subscale) touched almost half of the professionals with an interpretation of at least moderately exhausted, a higher rate than some previous findings in similar context [3]. These rates of burnout and psychological distress are likely to be related to the high number of confrontations with death and suffering reported by professionals. However, it appears that this high rate of confrontation is not incompatible with perceived benefits at both professional and personal levels, which certainly requires the ability to integrate these events into a broader reflection on the meaning of life, what really matters to us and the values that underpin our personal commitments.

Our study is the first to highlight the significant implication of the relationship that regular exposure to suffering and death modulates the intensity of burnout. The higher the degree of confrontation to suffering and death, the higher one’s depersonalization, which suggests that depersonalization may function as a defense mechanism—to put distance between oneself and the suffering of their patients [76]. This kind of depersonalization can be considered as the antithesis of empathy [77], that can be avoided or reduced (i) if the confrontation with suffering and death positively impacts the quality of the human relationship, (ii) if the meaning of work is important [26], and (iii) if the caregiver presents a higher agreeableness personality trait. Furthermore, coping strategies, years of professional experience [2] and line of service may also compensate for the increase in depersonalization. Our results are in line with previous linear regression results that have highlighted some protective factors against exhaustion such as the emotional stability personality trait [78], one’s sense of work meaning [26, 79], wellbeing, and the impact of confrontation on leisure activities. The main significant risk factor that has been highlighted so far is the conscientiousness personality trait, which can be interpreted as linked to a dysfunctional aspect of perfectionism [80].

Matching the results already described, the SEM path analysis presents a hierarchical, or integrative model, that takes into account simultaneously and quantitatively these different factors involved. Specifically, agreeableness and conscientiousness were found to correlate with self-esteem, which, together with death/suffering exposure positively impacting one’s personal representation of death, correlates with heightened work meaning [5]. Thus, one’s sense of work meaning has a direct positive link with wellbeing at work and is indirectly connected through one’s sense of accomplishment [26]. Finally, wellbeing at work, underpinned by this network of personal resources, is directly negatively linked to depression [81], which then demonstrates its direct impact on emotional exhaustion and anxiety levels. Taking one step further, we discover that the number of children is frequently linked to reduced work involvement with regard to fewer working hours and less frontline activity [82]. Therefore in consequence, rearing children (naturally coupled with higher age) is associated with a reduced degree of confrontation to suffering and death, having a direct impact in terms of a weaker depression-anxiety-exhaustion triad. These results suggest also the importance of finding an appropriate balance between family and work, and for future studies, the importance of taking into account the possible effects of life and career stages [83] which could also help to avoid Type I/II statistical errors.

As per the cluster analyses, a total of 109 health care professionals that vary in age and occupational group led to form three clusters that are more or less identical in size and very well-defined in model fit as per Fig. 2. All three clusters can be contrasted in respect to the juxtaposition of their pathological development and their professional resources experienced in the workplace. The health professionals in Cluster 2 experienced the most stress at work and had marked tendencies for pathological developments in terms of clinical depression, anxiety, or burnout states or symptoms. Unfortunately, they seemed to have less support and to procure only little energy and resources from their daily work. In contrast, the health professionals in Cluster 1 appear to experience an opposite reality at work. These individuals draw a lot of energy and resources from their work, and pathological developments are only rarely found in this cluster, which appears to follow in consequence. The two clusters differ above all in their meaning of work, and whether regular confrontation to suffering and death may have an alternative side, that is a positive impact in their life. Finally, the health professionals in Cluster 3 are more difficult to describe. In one respect, very few pathological developments are visible in this group, but apart from their sense of doing meaningful work and thus experiencing fulfillment, other positive work resources for them are sparse. As mentioned in the previous paragraph, as this cluster concerned older individuals who had children on average, we find lower degrees of confrontation to suffering and death, which may explain their weaker triad of depression-anxiety-exhaustion.

The SEM and cluster analyses certainly highlighted the important risk that regular confrontation to suffering and death can lead to burn-out and affective pathologies, but that crucially, the effect of this variable is moderated by the cognitive appraisal of its impact on one’s different domains of life. Thus, it seems that there is a work profile in the field of palliative and oncological care for which frequent confrontations to suffering and death do not represent a major obstacle to achieving personal growth and satisfactory relationships, nor interfere with their leisure activities and one’s sense of personal value [2]; honing in on said profile, by the way, may be useful for future student selection or employee screening programs. This cognitive restructuring, integrating one’s work experiences and challenges positively into personal life understandings, can contribute towards a meaningful life [4]. This notion is important to take into account in interventions aimed at addressing high rates of burnout or exhaustion in the field [83], not only for its prevention but also to promote wellbeing in health providers.

### Strengths of the study

An innovative aspect of this study concerns its integration both of the factors contributing to the wellbeing of professionals facing suffering and death, and the factors of psychopathological nature, such as of depression, anxiety, and burnout. In this perspective, the SEM approach is relevant in its capacity to analyze these parameters jointly and account for mediational effects through path analyses. In this respect, the study can also be said to be congruent with second wave positive psychology, which attempts to consider both the positive and negative in order to account for, at least in part, the complexity of human experiences [40, 84, 85].

So far, several studies have demonstrated some issues regarding the depersonalization and personal accomplishment scales of the MBI that combine with exhaustion in order to determine burnout positivity [86]. Thus, it may often be more insightful to explore Maslach’s three dimensions separately. The SEM approach realized herein offers the opportunity to understand their links with other risk and protection factors within a single, cohesive model. In this sense, the SEM alleviates the constraint of the separate linear regressions of the MBI subscales, and for which a total composite MBI score would not be interpretable. Moreover, another strength of this study lies in the data-driven approach employed in the different models that explores the phenomena without the confirmation bias associated with the typical counterfactual approach.

### Weaknesses of the study

The sample of participants in this study is small, but acceptable when compared to other studies in this population [87]. However, the participation rate may nevertheless represent a selection bias. It is therefore likely that caregivers affected by burnout may have been more motivated to respond to this survey, however of which, it is also possible that some may have exaggerated their burnout score for the purpose of making claims, as the study was formally presented to them in the framework of burnout during their departmental meetings. However, this bias does not call into question our interpretations of the regression, SEM, and cluster analyses, on the contrary: all of the analyses were data driven, only approximately five-percent of participants were automatically detected as outliers in the regressions, many rigorous model fit diagnostics were reported in the text for each approach, and the diagnostics were robustly satisfied in each case.

Another notable weakness is the validation of the CSDQ questionnaire created specifically for this study. Nevertheless, it must be pointed out that the development of the questionnaire was very structured and theory-based. In the first part, the results of systematic reviews were considered as a basis [5, 6]. The six questions in the second part and the five coping strategies in the third part were collected and summarized using the Delphi method [88] in the research team after interviews with different health professionals of the Oncology and Palliative Department. Furthermore, the coping subscale has a very low Cronbach’s alpha (0.48) and only includes social coping (colleagues/family), whereas many people turn to other sources of coping (leisure activities). As such, this scale is not generalizable in its present form and should be revised accordingly. It would then be necessary to test its validity.

From a medical and clinical point of view, this study should be taken with caution since the MBI scale is not a medical diagnosis per se. Moreover, just like the concept of burnout, this scale has been criticized lately particularly regarding its personal accomplishment and depersonalization subscales; we therefore made sure to also report its exhaustion subscale which is more directly related to burnout. Today, one of the most convincing models of burnout and work engagement is the Job Demands-Resources [89] model whose widely established results of a two-dimensional, reciprocal structure confirm the findings of this study [90]. In addition, our results require careful interpretation with regard to the causal link between the modeled factors. Only a longitudinal study with a medical diagnostic interview would strengthen the interpretations of the model described in this article. Finally, it should also be acknowledged that the entire organizational dimension of the stressors that lead to burnout was not the subject of this study.

### Meaning of the study: possible implications for clinicians

The integrative approach provided herein offers an important clinical angle for understanding the dynamics of mental disorders [91]. Designing models and interventions that include both positive and negative psychological variables provides a more comprehensive landscape of the factors involved, particularly those for preventive actions. Among the various variables considered in our study, our model highlights the importance of the meaning of work and the cognitive restructuring of stressors in accordance with the appraisal model [92]. Looking now at an interventional perspective, Acceptance and Commitment therapy (ACT), an intervention targeting the meaning and values of work, has been shown to be effective in reducing general distress work-related distress as indicated in a recent meta-analysis [93]. Another innovative way is the value-based meaning centered approach developed by Wong [94], which was also conceived to be implemented in the work context. This approach considers work at three different levels (individual, organization and society) and is based on an understanding of meaning from the PURE model (Purpose, Understanding, Responsibility and Enjoyment/Evaluation) [94]. This framework emphasizes a transition in motivation and attitudes from self-centeredness to self-transcendence. More recently, an online and inexpensive intervention focusing on writing down why work is meaningful has also been shown to be effective in increasing the meaning of work and work engagement among employees who participated [95, 96]. The advantage of such an intervention is that it does not require disproportionate means to be carried out and that it can represent a first step in an institution to reflect on the meaning given to work. Ideally, all these meaning-centered interventions have to include both individual components and organizational factors to buffer the negative impacts of a potentially traumatizing work context and strengthen wellbeing [98]. Finally, the informal peer support should be recognized and valued, as it allows for the sharing of experiences and an understanding of emotional experiences that are often not understood by people outside these particular professional contexts.

Thus, confrontation with the impermanence of life may have a positive and protective role for some people. In addition, when promoting wellbeing and resilience abilities in healthcare professionals, it seems essential to keep in mind the importance of the ecological approach. Therefore, particular attention should be given in the future to integrate both individual components and organizational/systemic factors to mediate the negative impacts of a potentially traumatizing context [97, 98].

## Conclusions

Our results show that burnout is not to be neglected, but it is not incompatible with a state of well-being. Being regularly confronted with death at work can lead to psychological distress and burnout, especially among the professionals working at the bedside who, because of their role, are exposed to confrontations for longer. However, it turns out that these confrontations can also bring professional and personal benefits. The representation of death and impermanence of life can, in contrast, be a catalyst by providing an opportunity to reflect on the values, meaning of work and even the meaning of life and thus constitute a beneficial and powerful source of well-being.

From an intervention perspective, the meaning attributed to work is a key factor both in preventing burnout and in determining well-being at work: interventions focusing on this dimension should therefore be a priority for healthcare professionals, but they should concern both the managerial and the individual levels. The results of this study also show that peer support deserves to be more widely recognised and valued. It is the most frequently cited form of support such as formal supervisions. It enables to share experiences and an understanding of the emotional experience that is often unrecognized by people not involved in these confrontations.

Finally, the implications of our modeling results can be further concretized in being linked back to the ancient “tetrapharmakos” principals of the Epicureans, in particular: “Don’t worry about death” and “What is terrible is easy to endure”. We can regulate our confrontations to the adversities that often hinder us, by the way in which we perceive them, which in turn, can ultimately be an instrumental source of wellbeing.

### Electronic supplementary material

Below is the link to the electronic supplementary material.

Supplementary Material 1

## Data Availability

The datasets generated and analysed during the current study are not publicly available due ethical and privacy reasons but are available from the corresponding author on reasonable request.

## Abbreviations

ACT: Acceptance and Commitment Therapy
AGFI: Adjusted Goodness-of-Fit
CSDQ: Confrontation to Suffering and Death Questionnaire
GFI: Goodness-of-Fit
HADS: Hospital Anxiety and Depression Scale
IPWW: Index of Psychological Wellbeing at Work
MBI: Maslach Burnout Inventory
MS: Work Meaning
RSE: Rosenberg’s Self-Esteem Scale
SEM: Structural Equation Modelling
TIPI: Ten Item Personality Index
