# Supplementary material for: Impact of confrontation to patient suffering and death on wellbeing and burnout in professionals: a cross-sectional study
Source: BMC Palliat Care. 2024 Mar 15;23:74. doi: 10.1186/s12904-024-01393-8 (PMC10941396; doi:10.1186/s12904-024-01393-8)

Impact of confrontation to patient suffering and death on wellbeing and burnout in professionals: a cross-sectional study

**Supplementary data**

**Figure S1**
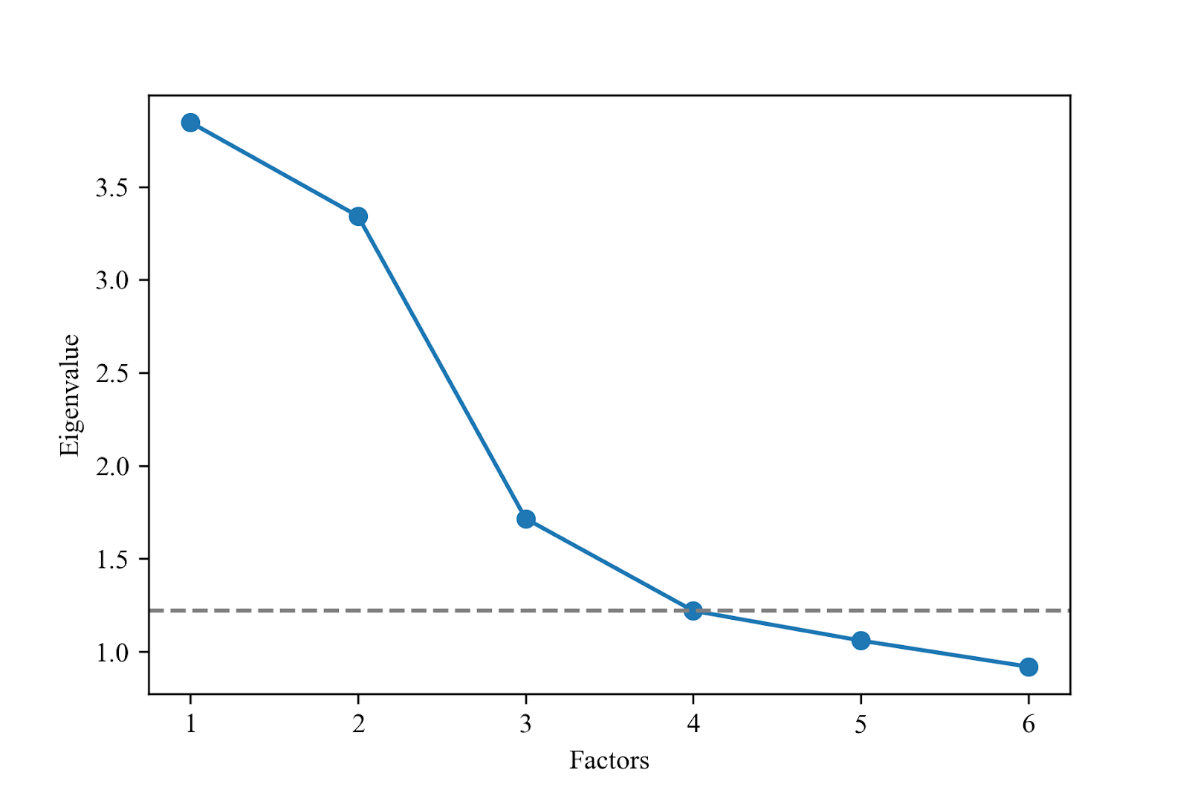


**Figure S1.** Scree plot analysis that supports the latent 3-factor structure of the CSDS. The dotted line reflects the “Elbow” method where the factors begin to diminish in a slow, linear fashion, suggesting that noise is predominantly being fit in these subsequent factors. (*n* = 109)

**Table S1.** Exploratory Factor Analysis (EFA) for the CSDS Scale (*n* = 109)

| **Subscale** | **Item Number** | **Confrontation** | **Impact** | **Coping** |
| --- | --- | --- | --- | --- |
| Confrontation |  |  |  |  |
|  | Q1 | 0.77 |  |  |
|  | Q2 | 0.68 |  |  |
|  | Q3 | 0.77 |  |  |
|  | Q4 | 0.65 |  |  |
|  | Q5 | 0.76 |  |  |
|  | Q6 | 0.81 |  | -0.24 |
| Impact |  |  |  |  |
|  | Q7 |  | 0.68 |  |
|  | Q8 |  | 0.64 |  |
|  | Q9 |  | 0.66 |  |
|  | Q10 |  | 0.54 |  |
|  | Q11 |  | 0.67 |  |
|  | Q12 |  | 0.67 |  |
| Coping |  |  |  |  |
|  | Q13 |  |  | 0.58 |
|  | Q14 |  | 0.32 | 0.48 |
|  | Q15 |  |  | 0.33 |
|  | Q16 |  | -0.23 | 0.49 |
|  | Q17 |  |  | 0.17 |
| SS Loadings |  | 3.39 | 2.75 | 1.09 |
| Explained Variance |  | 19.8% | 16.0% | 6.4% |
| n = 531 Participants |  | Total Explained Variance | | 42.4% |

Note: loadings below 0.20 in the non-relevant factor are not shown.

**Table S2.** Item Descriptive Statistics for the CSDS (*n* = 109)

| **Subscale** | **Item Number** | **Mean** | **SD** | **Skew** | **Kurtosis** | **ITC** |
| --- | --- | --- | --- | --- | --- | --- |
| Confrontation |  |  |  |  |  |  |
|  | Q1 | 2.31 | 1.2 | 0.3 | -1.4 | 0.71 |
|  | Q2 | 1.54 | 0.9 | 1.7 | 1.7 | 0.65 |
|  | Q3 | 2.5 | 1.1 | 0.2 | -1.2 | 0.72 |
|  | Q4 | 2.36 | 1 | 0.3 | -1.1 | 0.6 |
|  | Q5 | 2.77 | 1 | 0 | -1.3 | 0.69 |
|  | Q6 | 2.59 | 1 | 0.3 | -1.1 | 0.73 |
| Impact |  |  |  |  |  |  |
|  | Q7 | 2.76 | 3.1 | -0.1 | -0.4 | 0.6 |
|  | Q8 | 3.05 | 3.4 | -0.2 | -0.5 | 0.54 |
|  | Q9 | 1.06 | 3.3 | 0 | 0 | 0.61 |
|  | Q10 | 1.03 | 3.5 | 0.1 | 0 | 0.5 |
|  | Q11 | 3.61 | 3.5 | -0.7 | 0.6 | 0.6 |
|  | Q12 | 2.53 | 3.9 | -0.4 | -0.2 | 0.58 |
| Coping |  |  |  |  |  |  |
|  | Q13 | 2.54 | 1.4 | 0.6 | -0.6 | 0.35 |
|  | Q14 | 4.95 | 1.4 | -1.4 | 0.7 | 0.38 |
|  | Q15 | 3.83 | 1.9 | -0.1 | -1.6 | 0.26 |
|  | Q16 | 1.45 | 1 | 2.3 | 4.5 | 0.28 |
|  | Q17 | 1.21 | 0.8 | 4.9 | 25.1 | 0.09 |

.Note: *n* = 109

**Table S3.** *Summary table and guide for all variables* (*n* = 109)

| **Variable** | **Measure or Interpretation** | **Scale** | ***M (SD)* or Freq** |
| --- | --- | --- | --- |
| **Burnout (MBI)** | Burnout positivity based on the MBI subscales above | 0 \| 1 | 84.3% \| 15.7% |
| **Exhaustion (MBI)** | Degree of exhaustion at work | 0 to 54 | 25.8 (9.4) |
| **Depersonalization (MBI)** | Degree of depersonalization at work | 0 to 30 | 10.0 (4.9) |
| **Accomplishment (MBI)** | Degree of accomplishment at work | 0 to 48 | 45.5 (7.0) |
| **Anxiety (HADS)** | Degree of Anxiety | 0 to 21 | 6.8 (3.3) |
| **Depression (HADS)** | Degree to Depression | 0 to 21 | 3.7 (2.9) |
| **Confrontation (CSDS)** | Degree of confrontation to patient suffering and death | 6 to 24 | 14.1 (4.9) |
| **Coping (CSDS)** | Degree to which one seeks coping support | 5 to 30 | 14.0 (3.9) |
| **Impact (CSDS)** | Sum score of the CSDS impact variables below | -48 to 48 | 14.0 (14.9) |
| **Impact Death Rep (CSDS)** | Degree of impact on one’s representation of death | -8 to 8 | 2.5 (3.9) |
| **Impact Entourage (CSDS)** | Degree of impact on one’s work entourage or relations | -8 to 8 | 1.1 (3.3) |
| **Impact Leisure (CSDS)** | Degree of impact on one’s leisure activity or hobbies | -8 to 8 | 1.0 (3.5) |
| **Impact Life Rep (CSDS)** | Degree of impact on one’s representation of life | -8 to 8 | 3.6 (3.5) |
| **Impact Patient Rel (CSDS)** | Degree of impact on one’s patient relations | -8 to 8 | 2.8 (3.1) |
| **Impact Usefulness (CSDS)** | Degree of impact on one’s sense of work utility | -8 to 8 | 3.0 (3.4) |
| **Work Wellbeing (IPWW)** | Sum score of the IPWW variables below | 0 to 6 | 5.0 (0.6) |
| **Recognition (IPWW)** | Degree to which one finds their work is acknowledged | 0 to 6 | 4.7 (0.8) |
| **Competency (IPWW)** | Degree of work competency | 0 to 6 | 5.2 (0.6) |
| **Engagement (IPWW)** | Degree of engagement at work | 0 to 6 | 5.0 (0.6) |
| **Fulfillment (IPWW)** | Degree of fulfillment by one’s work | 0 to 6 | 5.0 (0.7) |
| **Interpersonal (IPWW)** | Degree of interpersonal relationship quality at work | 0 to 6 | 5.2 (0.6) |
| **Work Meaning (MS)** | Degree of meaning that one attributes to their work | 1 to 6 | 5.3 (0.7) |
| **Self-Esteem (RSE)** | Degree of self-esteem | 10 to 40 | 32.9 (4.2) |
| **Agreeableness (TIPI)** | Big-5 Agreeableness trait | 1 to 7 | 5.6 (1.0) |
| **Conscientiousness (TIPI)** | Big-5 Conscientiousness trait | 1 to 7 | 6.2 (0.8) |
| **Emotional Stability (TIPI)** | Big-5 Emotional Stability trait | 1 to 7 | 4.9 (1.2) |
| **Extraversion (TIPI)** | Big-5 Extraversion trait | 1 to 7 | 4.2 (1.4) |
| **Openness (TIPI)** | Big-5 Openness trait | 1 to 7 | 5.2 (1.0) |
| **Line of Service** | 1st, 2nd, or 3rd Line of Service | 1, 2, 3 | 1.6 (0.7) |
| **Prof Experience** | Ordinal, 1: <5, 2: 5-10, 3: 11-16, 4: 17-22, 5: >23 years working since diploma | 1 to 5 | 3.2 (1.4) |
| **Years Working** | Continuous, # years working in current healthcare job | 0 to 40 | 4.4 (4.9) |
| **Work Hours** | Ordinal, 1: 20-30, 2: 31-40, 3: >40 work hours per week | 1 to 5 | 2.6 (1.0) |
| **Age** | Ordinal, 1: 19-29, 2: 30-39, 3: 40-49, 4:50-59, 5: >60 y.o. | 1 to 5 | 2.6 (1.0) |
| **Gender** | Binary, Female, Male (coded 1) | 0 \| 1 | 79% \| 21% |
| **Cohabitation** | Binary, currently living with a romantic partner (coded 1) | 0 \| 1 | 30% \| 70% |
| **N Children** | Continuous, Number of children | 0 to 6 | 0.8 (1.1) |
|  | | | |

**Table S4.** *Observed Fit Indices and Appropriate Thresholds for the Structural Equation Model* (*n* = 109)

| Indices | Observed Value | Acceptable Threshold |
| --- | --- | --- |
| Model χ² / *df* | 1.12 | < 5.0 |
| Comparative Fit Index (CFI) | 0.96 | > 0.90 |
| Incremental Fit Index (IFI) | 0.96 | > 0.90 |
| Tucker Lewis Index (TLI, a.k.a. NNFI^1^) | 0.95 | > 0.90 |
| Root Mean Squared Error of Approximation (RMSEA) | .036 | < 0.10 |
| RMSEA 90% Confidence Interval | [0.00; 0.061] | [0.00; Close to RMSEA] |
| RMSEA *p*-value (close fit^2^) | .797 | > 0.10 |
| Goodness of Fit Index (GFI) | 0.82 | > 0.90 |
| Adjusted Goodness of Fit Index (AGFI) | 0.75 | > 0.90 |
| Model χ² (*df* = 154)^3^ | 173.40, *p* = .14 | *p > .05* |
| Baseline or null model χ² (*df =* 180) | 665.23, *p < .001* | *p < .05* |
| *Note:* 1. Non-normed fit index. 2. Here large *p*-values (i.e., *p* > 0.10) reflect a close fit; 3. Minimum function test statistic: null hypothesis corresponds to an ideal model, thus smaller χ² values, and hence *p*-values > .05 are preferred for the observed model. | | |

**Table S5.** *Two-sample t-tests (cluster vs. cluster) for the variables included in Figure 2 (test statistic t values and significance *p<.05 ; **p<.01 ; ***p<.001, n* = 109*)*

| **Variable Cluster:** | **1 - 2** | **1 - 3** | **2 - 3** |
| --- | --- | --- | --- |
| **Fulfillment (IPWW)** | 11.95*** | 5.69*** | -7.54*** |
| **Impact Life Rep (CSDS)** | 9.36*** | 8.17*** | -1.72 |
| **Work Meaning (MS)** | 8.95*** | 3.51*** | -5.93*** |
| **Competency (IPWW)** | 7.42*** | 2.80** | -5.46*** |
| **Engagement (IPWW)** | 7.23*** | 3.86*** | -4.71*** |
| **Recognition (IPWW)** | 6.83*** | 2.57* | -6.05*** |
| **Interpersonal (IPWW)** | 6.38*** | 4.15*** | -3.33** |
| **Accomplishment (MBI)** | 6.19*** | 3.83*** | -3.29** |
| **Impact Entourage (CSDS)** | 6.03*** | 4.61*** | -2.74** |
| **Impact Death Rep (CSDS)** | 5.97*** | 6.21*** | -0.69 |
| **Self-Esteem (RSE)** | 5.67*** | 1.08 | -5.14*** |
| **Impact Patient (CSDS)** | 5.63*** | 6.78*** | 0.01 |
| **Impact Leisure (CSDS)** | 5.21*** | 4.12*** | -1.46 |
| **Impact Usefulness (CSDS)** | 5.06*** | 5.62*** | -0.28 |
| **Conscientiousness (TIPI)** | 4.19*** | 2.74** | -2.13* |
| **Extraversion (TIPI)** | 3.30** | 2.06* | -1.57 |
| **Openness (TIPI)** | 3.05** | 3.05** | -0.32 |
| **Emotional Stability (TIPI)** | 2.93** | 1.43 | -1.88 |
| **TIPI Agreeableness (TIPI)** | 2.65* | 2.17* | -0.94 |
| **Depersonalization (MBI)** | -4.00*** | 0.07 | 4.52*** |
| **Anxiety (HADS)** | -4.01*** | -1.06 | 3.56*** |
| **Exhaustion (MBI)** | -4.28*** | -0.48 | 4.87*** |
| **Depression (HADS)** | -6.93*** | -0.03 | 7.67*** |
| **Degree of Confrontation (CSDS)** | -1.11 | 1.14 | 2.52* |
| *Note:* Variables were Yeo-Johnson transformed to approximate a normal distribution. Positive values indicate larger values in the first cluster listed in the column. In the interest of brevity, only variables with significant differences are provided in the table. | | | |

**Table S6.** *Confrontation, coping and impact scale (CSDQ) with item quotation* **(***n =* 109)


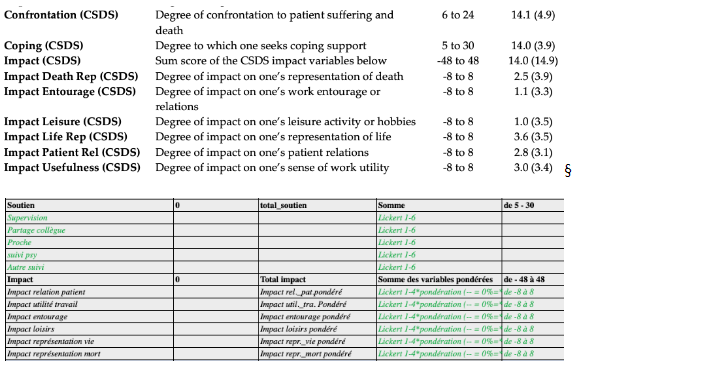

Supplement: Supplementary file 1 — Supplementary Material 1 [file 12904_2024_1393_MOESM1_ESM.docx]
